# Lower-crustal earthquakes in southern Tibet are linked to eclogitization of dry metastable granulite

**DOI:** 10.1038/s41467-018-05964-1

**Published:** 2018-08-28

**Authors:** Feng Shi, Yanbin Wang, Tony Yu, Lupei Zhu, Junfeng Zhang, Jianguo Wen, Julien Gasc, Sarah Incel, Alexandre Schubnel, Ziyu Li, Tao Chen, Wenlong Liu, Vitali Prakapenka, Zhenmin Jin

**Affiliations:** 1 State Key Laboratory of Geological Processes and Mineral Resources, School of Earth Sciences, China University of Geosciences, 430074 Wuhan, China; 2 Center for Advanced Radiation Sources, The University of Chicago, Chicago, IL 60439 USA; 3 Department of Earth & Atmospheric Sciences, Saint Louis University, St. Louis, MO 63108 USA; 4 Center for Nanoscale Materials, Argonne National Laboratory, Argonne, IL 60439 USA; 5 Laboratoire de Géologie, CNRS UMR 8538, École Normale Supérieure PSL Research University, Paris, 75231 France

## Abstract

Southern Tibet is the most active orogenic region on Earth where the Indian Plate thrusts under Eurasia, pushing the seismic discontinuity between the crust and the mantle to an unusual depth of ~80 km. Numerous earthquakes occur in the lower portion of this thickened continental crust, but the triggering mechanisms remain enigmatic. Here we show that dry granulite rocks, the dominant constituent of the subducted Indian crust, become brittle when deformed under conditions corresponding to the eclogite stability field. Microfractures propagate dynamically, producing acoustic emission, a laboratory analog of earthquakes, leading to macroscopic faults. Failed specimens are characterized by weak reaction bands consisting of nanometric products of the metamorphic reaction. Assisted by brittle intra-granular ruptures, the reaction bands develop into shear bands which self-organize to form macroscopic Riedel-like fault zones. These results provide a viable mechanism for deep seismicity with additional constraints on orogenic processes in Tibet.

## Abstract (web summary)

The triggering mechanism of deep seismicity in Tibet remains unclear. Here the authors use experiments to show that granulite when deformed becomes brittle as it passes into the ecologite stability field developing macroscopic riedel fault zones thus providing an explanation for deep seismicity in Southern Tibet.

## Introduction

**Fig. 1:**
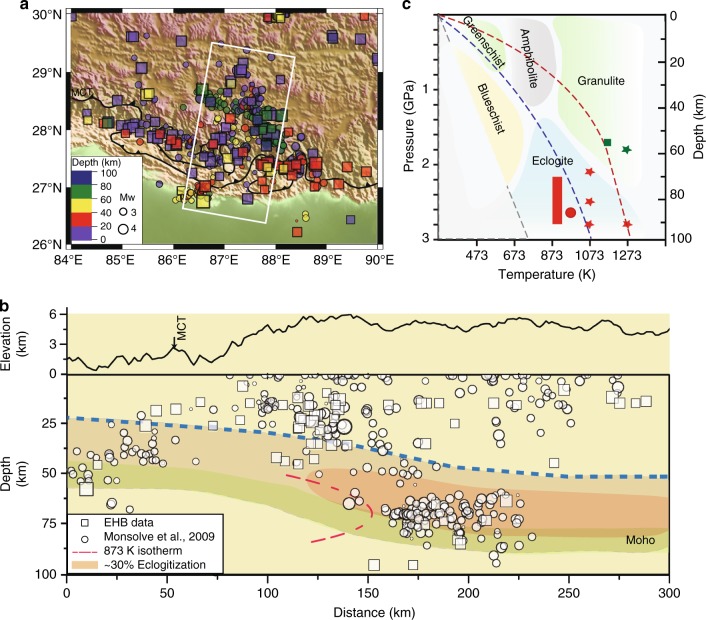
Seismicity in southern Tibet and the relation to granulite-eclogite metamorphic reaction. **a** A map view of the area of interest with earthquake locations from the EHB Bulletin (http://www.isc.ac.uk/ehbbulletin/) (relocated using teleseismic data) and from relocation^14^ using the Himalaya Napel Tibet Seismic Experiment^18^ data. **b** A schematic cross-section view normal to the arc of the Main Central Thrust with earthquake hypocenters shown in **a**. The blue dashed line marks the approximate boundary between the Tibetan crust (above) and the subducted Indian crust (below). The range of the Moho depths is indicated by the greenish band, whose upper, and lower bounds are from^14^ and^57^, respectively. “873 K isotherm” from thermokinematic modeling^57^ and the “30% eclogitization” region based on received function models^32^ are indicated. **c** A schematic phase diagram of granulite-facies rocks in relation with other facies (modified from^70^). Estimated P-T conditions of xenolith samples are given as the following: eclogite—red rectangle^36^ and red circle^37^; granulite—green squares^31^. Experimental conditions are given as color-coded stars. Red symbols are in eclogite field and green in granulite field

Tibetan Plateau, the largest mountain range on Earth, is formed by the collision between Indian and Eurasian continents since ~50 million years ago, resulting in significant deformation in central and eastern Asia with a remarkably thickened crust in southern Tibet (Fig. 1a)^1–3^ and pushing the Mohorovičić seismic discontinuity (Moho) to ~80 km deep^1,4–6^. The underlying geodynamic process has been under intense debate for decades, a central issue being the mechanical nature of subducted Indian crust and the underlying upper mantle^3,7,8^. Earlier seismic studies report bimodal depth distributions of seismicity, interpreted as an aseismic and weak lower crust sandwiched between the seismogenic upper crust and the upper mantle^9^. Contrary to this “jelly sandwich” model, the “crème brûlée” model argues for strong upper and lower crust overlying a weak upper mantle^10^. The two models have drastically different implications in thermal structure, mass transfer, water storage, and melt distribution in the lower crust and upper mantle. While the “jelly sandwich” model requires water and/or partial melt enrichment^7,11^, suggesting lateral channel flow of materials within the mid-crust to compensate the extra surface load imposed by the Himalaya mountain range^12^, the “crème brûlée” model suggests compensating flows occurring primarily in the upper mantle^13^.

Seismicity is the most direct indicator of mechanical state of Earth’s interior. Most recent studies show that seismicity distribution in southern Tibet is more or less continuous from the surface to as deep as 100 km (Fig. 1b)^14–17^. While the debate on crust and mantle strength continues [e.g., see reviews in^11,18^], one must address another crucial issue, that is, how can earthquakes occur in the lower crust and upper mantle, which are well below the brittle-ductile transition depth^19^, with plastic yield strength of rocks well below that of Byerlee’s friction^20^. As far as the lower crust is concerned, three hypotheses have been proposed. The first is a thermal runaway process where a self-amplifying mechanical instability arises from the combination of shear localization and grain-size reduction within a visco-plastic material^21^. Crucial issue here is the conditions under which shear localizations nucleate and self-amplify, leading to failure^22^. Such conditions are poorly known and just begun to be investigated in the laboratory^23^. The second is dehydration embrittlement, during which a metamorphic dehydration reaction has the potential to raise pore pressure, thereby lowering the effective pressure, permitting brittle failure^24^. This hypothesis requires sufficient structural water in constituent minerals and is considered a good candidate for seismicity in subducted oceanic low crust^25,26^. However, xenolith samples from Tibet suggest that the Indian lower crust lacks hydrous minerals^27^, making this mechanism less likely to operate. The third is eclogitization-induced instability^28,29^. The Indian lower crust consists predominantly of granulite-facies rocks^30,31^, which become metastable when entering the eclogite-facies stability field through subduction, due to the sluggish reaction kinetics within these nominally anhydrous rocks (Fig. 1c). Seismic and gravity anomaly data support the existence of eclogite in subducted Indian crust^2,32–35^ and eclogite xenoliths have been found in central Tibet^36,37^. Pseudotachylytes, usually formed by brittle failure followed by frictional melting during shallow seismicity, are found in exhumed deep continental rocks in western Norway^38^ where dominantly granulite-facies rocks contain eclogite-facies minerals in close association with the pseudotachylytes, suggesting that granulite-facies rocks transformed partially at depths greater than 60 km, under conditions corresponding to the eclogite-facies stability field. Some suggest that deep crustal seismicity under southern Tibet may be analogous to that in western Norway^29^. Others, however, argue that eclogitization involves reconstructive phase transformations in constituent minerals and is unlikely to trigger mechanical instability^24^.

With the advent of new experimental techniques^39,40^, a quantitative investigation is now possible to address the debate on eclogitization-induced instability in the laboratory. It has been shown that eclogitization of lawsonite-blueschist (a metabasalt abundant in oceanic crust) produces mechanical instability^41^. Here we investigate experimentally the mechanical behavior of dry granulite under various pressure and temperature conditions within both granulite and eclogite stability fields, to examine the micromechanics responsible for rupture nucleation and self-organization leading to macroscopic faulting. By combining synchrotron x-ray diffraction and imaging for stress and strain measurements with acoustic emission (AE) monitoring, we show that eclogitization of metastable granulite indeed induces mechanical instability due to formation of fine-grained weak reaction bands. Using advanced seismological tools, we locate the AE events, and track fracturing process in both space and time. Such four-dimensional (4-D) information, along with microstructural analysis on recovered samples after failure, provides a unique opportunity to evaluate the mechanism of eclogitization-induced instability for rupture nucleation and propagation in the continental lower crust.

## Results

### Samples and experimental setup

The starting materials were synthetic granulite by mixing hand-picked natural minerals, 65% plagioclase (Plg), 20% quartz (Qtz) and 15% clinopyroxene (Cpx) by weight. In molar fractions, Plg was ~52% albite (Ab) and 48% anorthite (An), and Cpx was ~66% diopside (Di), 22% ferrosilite, and 12% wollastonite (Supplementary Table 1). The mixture was sintered at 2 GPa and 1273 K (in the stability field of granulite-facies) or 3 GPa and 1073 K (in eclogite-facies field), respectively, for 6 h, yielding a series of pure granulite (denoted by G) and partially eclogitized granulite (EG) samples, respectively. Average grain size was about 20–50 μm. The degree of eclogitization in the EG samples was limited to <10% in volume and the reactions were confined to vicinities adjacent to grain boundaries (Supplementary Fig. 1). Average whole-rock water contents were < 280 ppm (Supplementary Fig. 2, Supplementary Table 2), about half those reported as “pre-dried” granulite samples in^42^.

Triaxial deformation experiments were carried out in both a modified Griggs apparatus (Supplementary Fig. 3)^42^ and a deformation-DIA (D-DIA) apparatus^40^. The latter was interfaced with synchrotron X-ray diffraction and imaging, as well as AE monitoring, providing vital real-time information critical for investigating faulting mechanism in-situ (Supplementary Figs. 4–5). Samples were deformed under pressure and temperature conditions corresponding to depths up to 90 km (Fig. 1c, Table 1).

**Table 1:** Experimental conditions, mechanical behavior, and AE activities

Expt ID	Initial Sample state	*P* (GPa)	*T* (K)	Strain rate (s^−1^)	Peak stress (MPa)	Max. strain %	Rupture style	No. of triggered AE events	Phase assembly
D1784	G	1.8	1273	1.2 × 10^−4^	150	23.9	Ductile	0	G^a^
D1995	G	2.1	1073	5.0 × 10^−5^	586	40.2		0	G
D1996	G	2.8	1073	7.0 × 10^−5^	2300	40.6	Macroscopic faults	956	G + Grt + Omp + Ky
D1997	G	2.8	1073	5.0 × 10^−5^	1500	42.0		198	G + Grt + Omp + Ky
D1787	EG	2.5	1073	1.0 × 10^−4^	366	24.2		531	G + Grt + Omp + Ky
GA125^b^	EG	3.0	1273	2.0 × 10^−4^	~500	40.2		n/a	G + Grt + Omp + Ky
D1736^c^	EG	2.8	1273	1.5 × 10^−4^	265	26.5	No macroscopic faults	148	G + Grt + Omp + Ky
D1738^c^	EG	2.8	1273	1.4 × 10^−4^	306	26.6		184	G + Grt + Omp + Ky

G pure granulite, EG partially eclogitized granulite

^a^Initial granulite phase assembly Plg + Cpx + Qz

^b^Experiment conducted in Griggs apparatus without AE monitoring. Stress estimated from Ref (42) under similar conditions

^c^Deformation stopped before reaching macroscopic failure

### Mechanical data and AE activity

The two G samples (D1784 and D1995) deformed in the granulite-facies field exhibit ductile behavior with no AEs observed. This is consistent with previous reports that granulite deforms primarily by dislocation creep under similar conditions^42,43^. In contrast, all the samples, both G and EG types, deformed in the eclogite-facies field, radiated numerous AEs. The brittle response is therefore clearly due to eclogitization of metastable granulite.

**Fig. 2:**
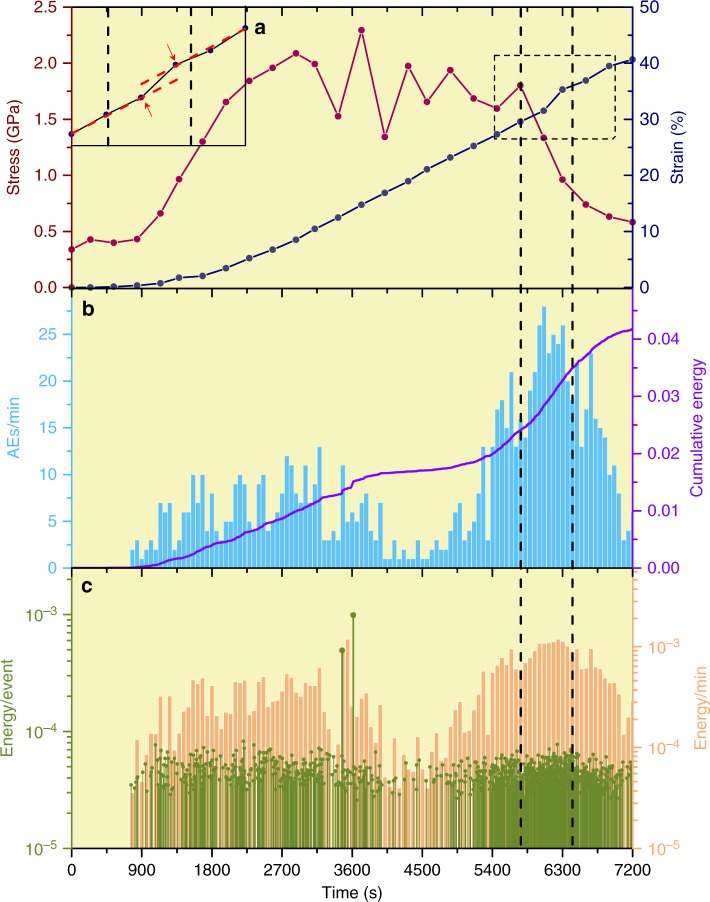
History of mechanical behavior and AE activity of a G sample (D1996) during deformation. **a** Differential stress and strain. Note large fluctuation in stress between 2700 and 5400 s. At macroscopic failure (centered around 6000 s, between two vertical dashed lines), a sudden jump in strain is observed (dashed box), associated with a > 50% stress drop. Inset is enlarged portion of the dashed box, showing the strain jump. **b** AE rates (in # per min) and cumulative energy release (in arbitrary units) during deformation. Note the two broad burst episodes and the peak centered around 6000 s, which corresponds to the strain jump and stress drop in **a**. **c** Individual relative AE energy (in arbitrary units) and energy release rate. Only two events near 3600 s are significantly large than the rest. The rest of the AE events have similar magnitudes, corresponding to M_W_ around −8.4–8.8^45^. Energy release rate also peaked around 6000 s. Vertical dashed lines mark the time window in which the AE activity peak (**b**, **c**) coincides with the strain jump during the stress drop (**a**)

Figure 2 summarizes the mechanical behavior of a G sample (D1996) deformed at 2.8 GPa and 1073 K. Deformation began at a differential stress of 0.3 GPa and axial strain started increasing at ~500 s. The sample was continuously deformed for 2 h, ending at ~40% strain. Differential stresses were high with abrupt fluctuations (Fig. 2a). AEs began appearing immediately after differential stress started increasing (Fig. 2b, c) and AE activities were episodic. Two broad burst episodes are clearly distinguished, separated by a quiet period. As will be discussed below, the two episodes correspond to the nucleation and growth of two large branches of macroscopic faults. The first episode ended with 2 large events (Fig. 2c), which were likely responsible for the large stress fluctuation near ~3600 s (Fig. 2a), although no clear strain discontinuity was produced. The second broad episode began around 5000 s, with higher AE rates. The largest burst of AE rate occurred around 6000 s (Fig. 2a, b), associated with a rapid stress drop and a visible discontinuity in the strain-time curve in Fig. 2a, probably due to macroscopic faulting. Amplitudes of AE events are typically 10–30 times greater that the triggered threshold (Supplementary Fig. 6), so relative radiated energies can be estimated based on areas under rectified signal envelope (Supplementary Fig. 7). The steepest cumulative energy release is associated with the second episode, ending with macroscopic failure (Fig. 2c).

**Fig. 3:**
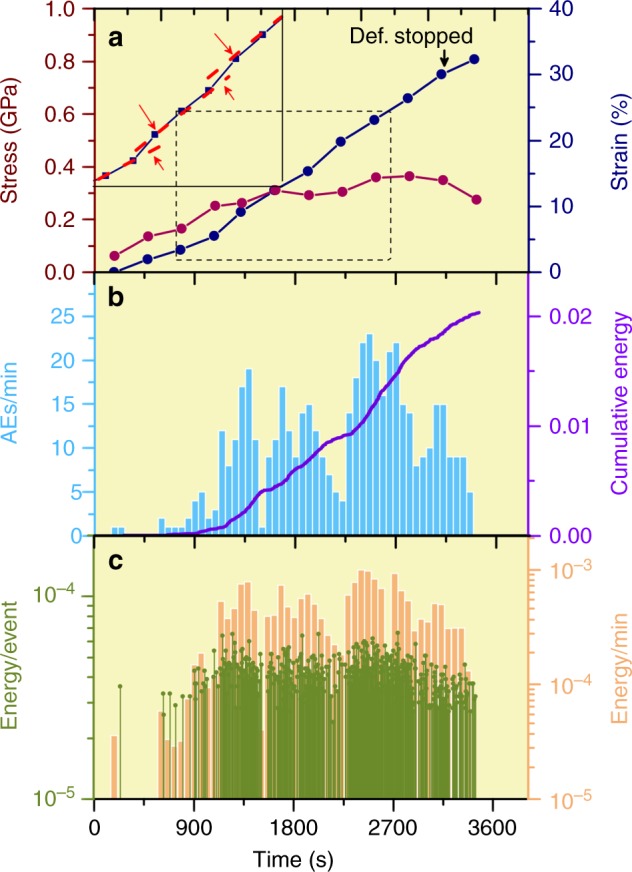
History of mechanical behavior and AE activity of an EG sample (D1787). **a** Differential stress and strain. A portion of the strain history (dashed box) is enlarged in the inset, showing two discontinuities. The decrease in stress near the end is due to termination of deformation, not to macroscopic failure. **b** AE rates and cumulative energy. Note episodic nature of the events, similar to that in Fig. 2. AE rate drops off rapidly after termination of deformation. **c** Individual event energy and energy release rate. Event energies are of similar magnitudes to those in Fig. 2

For the EG sample D1787, differential stresses were on the order of 0.4 GPa (Fig. 3a). Fluctuations of differential stress are also associated with strain jumps, however uncertainties and poor time resolution (~500 s) in stress measurements do not permit a more detailed examination. The sample was continuously shortened for ~1 h, reaching ~25% strain, where deformation was intentionally stopped before reaching ultimate failure. AE activities also began at very low strains and displayed an episodic behavior. The first large AE burst episode began around 1300 s (Fig. 3b, c), causing a discontinuous strain jump, probably due to macroscopic faulting (Fig. 3a). Another strain jump occurred near 2250 s, shortly before the largest peak of AE rate. AE activity decayed rapidly after the termination of deformation.

Mechanical responses of the two EG samples (D1736, 1738) at 1273 K are summarized in Supplementary Fig. 8. AE activity began only when bulk sample strains reached ~10–15 % and at flow stresses ~5% below the peak values (~0.3 GPa). With further increase in strain, AE activity increased rapidly. Flow stress peaked at 15–20% strain and then decreased. Deformation was stopped when flow stress dropped to ~2/3 of the critical value. Both stress and AE activity decayed rapidly due to stress relaxation, while strain remained constant within measurement errors, indicating that deformation was largely stopped.

### Tracking AE events in 4-D

**Fig. 4:**
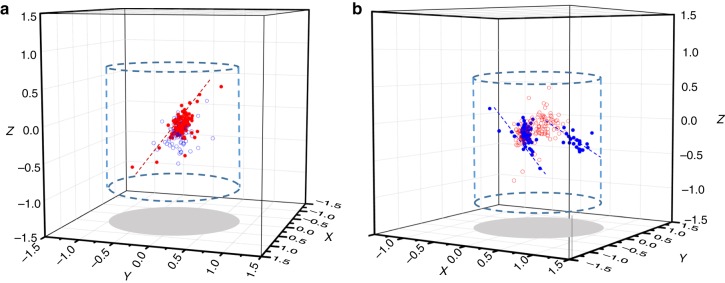
Projections of AE locations in D1996 showing near planar distributions. Blue dashed cylinders outline the sample after the experiment. **a** Group 1 events (red solid circles) form a planar distribution as indicated by the red dashed line (see Supplementary Movie 1 for history of event development). Group 2 events are shown as open blue circles in this projection. **b** Group 2 events (blue solid circles) form 2 planar distributions indicated by the blue dashed lines (see Supplementary Movie 2 for history of event development). Group 1 events are shown as open red circles in this projection. Scales shown in mm

For each pair of triggered events, their differential arrival times at each of the six transducers were measured using waveform cross correlation (CC) and were used to determine their relative location with the double-difference algorithm HypoDD^44,45^. Events were divided into several groups according to waveform similarity defined by correlation coefficients greater than 0.8. All the events were located within the samples that were analyzed. Spatial distribution of events in two largest groups in D1996 is shown in Fig. 4. Both groups initiated around the center of the sample, but later defined clearly distinguishable planar features, representing conjugated faults. Supplementary Movies 1 and 2 display the two groups of events in both space and time. All Group 1 events occurred within the first burst episode (Fig. 2b, c). Earlier events (600–1800 s) showed no clear preferential distribution in space, suggesting an early stage of microrupture nucleation. Events after 1800 s began forming a planar distribution, indicating the formation of a fault plane (Supplementary Movie 1). Group 2 events, in contrast, all occurred within the second burst episode and fell into two subgroups in space. The first subgroup was located in the center of the sample coinciding with many Group 1 events, and developed a planar branch after 6600 s. The second subgroup began forming after 5400 s, and, as it grew, developed into a better defined planar distribution (Supplementary Movie 2). One projection is shown in Fig. 4b, displaying the two fault-like distributions. The sample remained intact until ~6000 s, when the large strain discontinuity occurred shortly after the stress drop (Fig. 2a), and failed shortly after.

In the EG sample (D1787), whose deformation was stopped before reaching macroscopic failure, one large group (Group 1) dominated the AE activity, with more than 400 events, which also show near planar formations (Supplementary Movie 3). These planar features are less well-defined than those in D1996.

### Microstructural observations

**Fig. 5:**
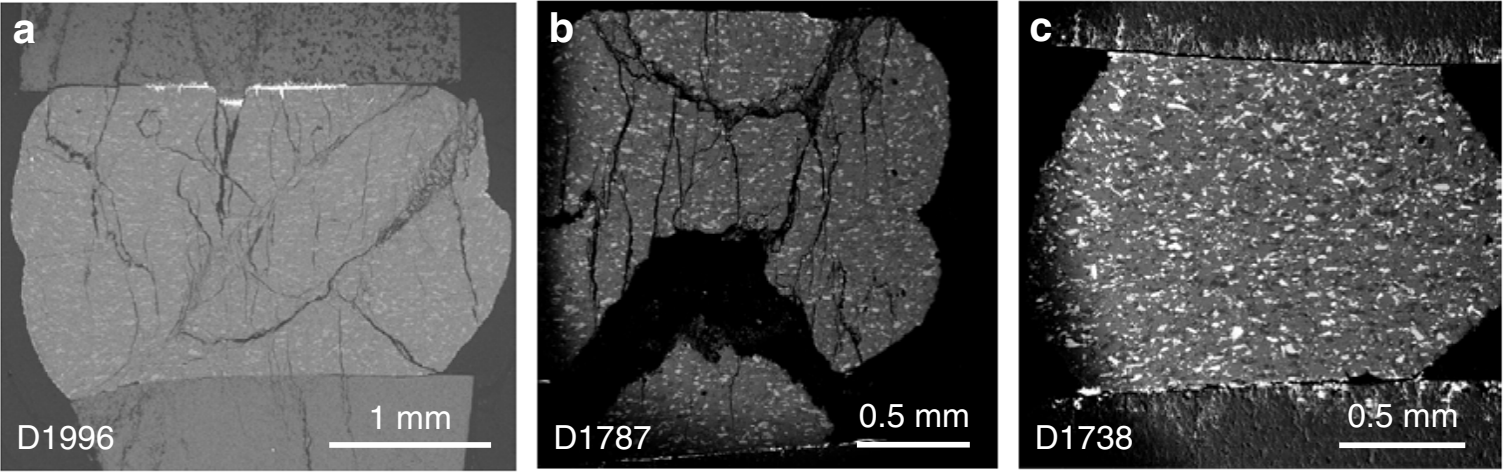
Low-magnification SEM images showing microstructure samples deformed under various conditions. **a** D1996 (G sample, 1073 K, to 40% strain). **b** D1787 (EG sample, 1073 K, to 30% strain). **c** D1738 (EG sample, 1273 K, to 30% strain. Both 1073 K samples contain through-going faults (**a**, **b**), whereas the 1273 K sample has no visible faults at this magnification

**Fig. 6:**
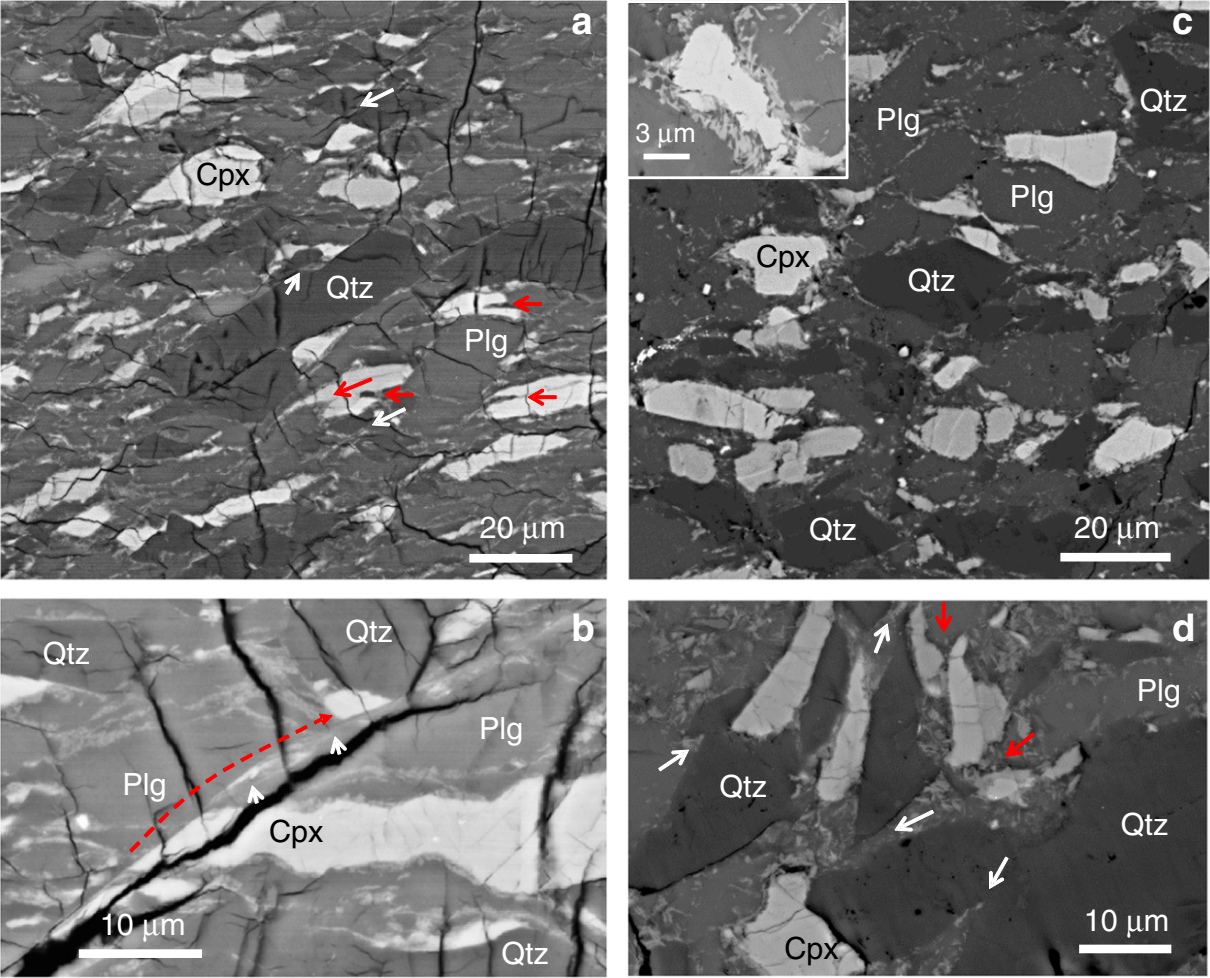
SEM micrographs of samples deformed at 1073 K. All images are oriented with the shortening direction vertical. **a**, **b** D1996 (G sample). **a** Note predominant NE-SW lighter linear features (NRBs) along grain boundaries which are filled with nm sized reaction products. Many grains are torn open with small “notches”. Red and white arrows indicate some notches in Cpx and Qtz, respectively. **b** A Cpx grain has been sheared off. Red dashed arrow show that the broken-off portion has a displacement of ~20 μm. A thin trace of reaction products can be seen connected the two parts of the grain. **c**, **d** D1787 (EG sample). **c** More reaction products are along Cpx grain boundaries compared to **a**. Inset shows details of reaction rim around a Cpx grain. The needle-shaped reaction grains are Ky. **d** Cpx and Qtz grains are frequently associated with triangular shaped Plg wedges filled with reaction products (red and white arrows point to wedges in Cpx and Qtz grains, respectively)

**Fig. 7:**
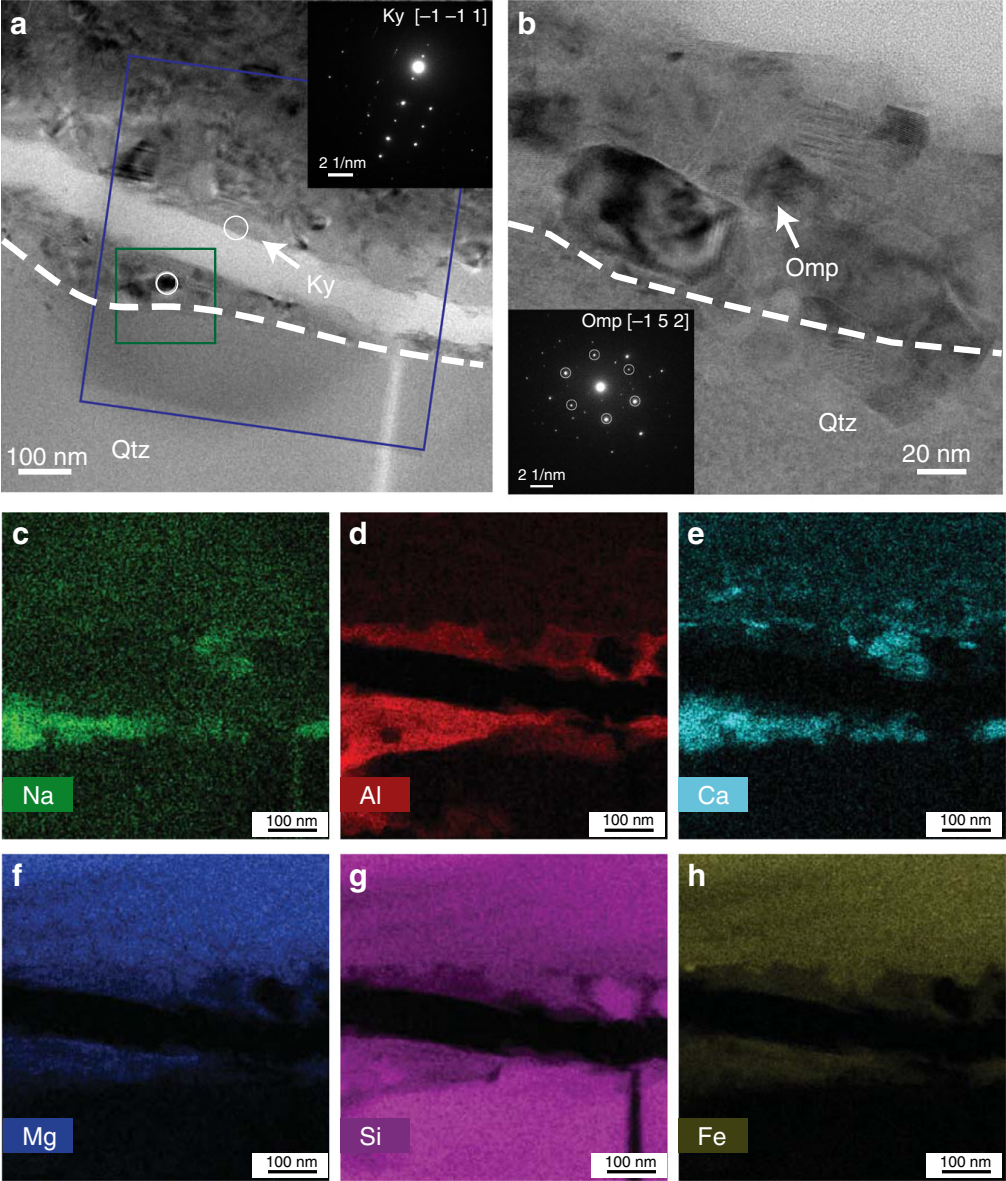
TEM micrographs and element distribution maps along a fault zone in D1996. **a** An NSB (above the dashed line) consisting of nano-grained reaction products, majority of which are less than 50 nm. One Ky grain is identified at the edge of the crack in the fault zone by the inset SAED pattern. **b** Enlarged area of the green box in **a**, where an Omp grain about 30 nm is identified by SAED (inset). No amorphous materials are found. **c**–**h** Element distribution maps of the area indicated by the blue box in **a**. Omp appears below the crack at the Qtz grain boundary, showing characteristic enrichment in Na, Al, and Ca. Ky appears above the crack, enriched in Al but depleted in Na and Ca. This FIB foil was cut from the location indicated by the short red line in Supplementary Fig 9

Scanning electron microscopy (SEM) shows that, at mm scale, both G and EG samples deformed at 1073 K display macroscopic faults (Fig. 5a, b), while the EG sample deformed at 1273 K contain no visible large faults (Fig. 5c). Figure 6 compares microstructures of the G (D1996) and EG (D1787) samples, both deformed at 1073 K. In both cases, eclogitization products are observed along grain boundaries and within Plg grains (Fig. 6a, c). Transmission electron microscopy (TEM), selected area electron diffraction (SAED), element mapping using energy-dispersive spectroscopy (EDS), and micro-Raman spectroscopy show that the reaction products are omphacite (Omp), kyanite (Ky), and garnet (Grt) (Fig. 7, Supplementary Figs. 9–12). No hydrous minerals, such as epidote, were detected.

**Fig. 8:**
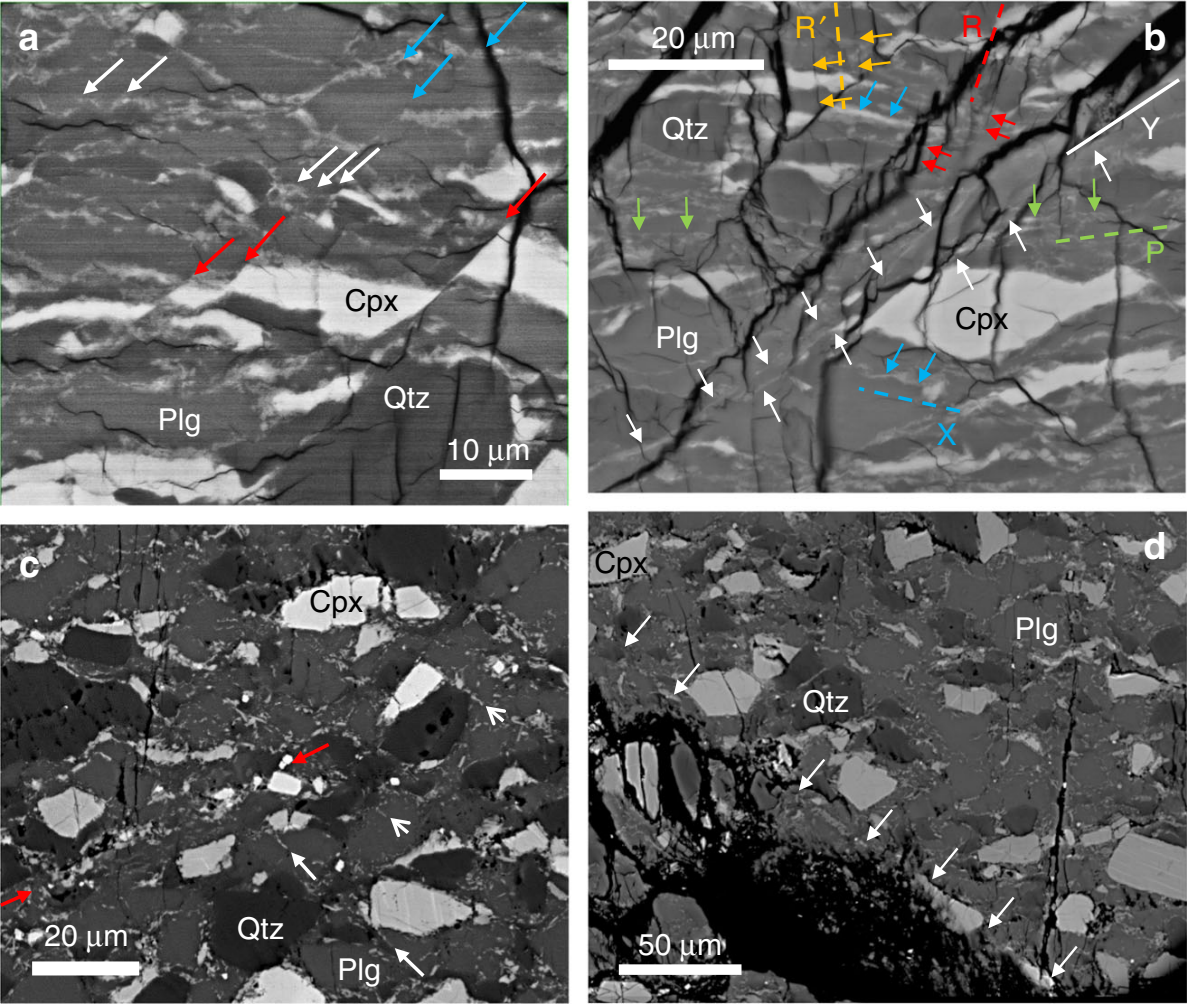
Organization of fault zones in samples deformed at 1073 K. **a**, **b** D1996 (G sample). **a** Wide-spread intra-grain ruptures. Red, blue, and white arrows mark intra-grain ruptures in Cpx, Plg, and Qtz, respectively. These ruptures are connected by thin NRBs to form larger, complex shear zones. **b** Formation of larger fault zones. Shear and ruptures developed along several directions, which can be interpreted as Riedel shears^52^. Y shears (solid white line; parallel to the main shear zone) dominate the microstructure, characterized by long and continuous NRBs cutting through multiple grains (delineated by white arrows). R and P shears are ~±30° from the Y shears (light green and red dashed lines and arrows). R′ and X shears are ~±60° from the Y shears (yellow and blue dashed lines and arrows). R and R′ shears are less well developed and commonly associated with microfractures. **c**, **d** D1787 (EG sample). **c** Wide-spread eclogitization reaction along grain boundaries and within Plg grains. Numerous reaction bands terminate within the Plg grains, along the NW-SE (white arrows). These reaction bands typically initiate from grain-grain contacts, suggesting a stress assisted reaction. Indicated in between the two opposing red arrows is a complex shear zone with multiple subparallel NRBs. Compared to D1996, D1787 contains numerous small voids along grain boundaries. These voids may be cavities caused by the large volume reduction of the reaction, although we cannot completely rule out the possibility of small grains popping off during polish. **d** A large shear band consisting of reaction products cutting through multiple grains with different mineralogy (delineated by white arrows that are nearly perpendicular to the band

In the G sample D1996 (Figs. 6a, b, 7, 8a, b, Supplementary Fig. 10), reaction products appear as continuous thin bands, along essentially all boundaries between different minerals. Within Plg grains, thin bands are wavy, densely spaced, and subparallel (Fig. 6a, b). Within these bands, grain sizes are extremely small, on the order of 50 nm (Fig. 7, Supplementary Fig. 10). These bands are hereafter referred to as nano-reaction bands (NRBs), as they consist of the above mentioned reaction products. Short notch-like openings are often observed along the boundaries of Cpx and Qtz grains (arrows in Fig. 6a). These “notches”, filled with Plg or reaction products, are likely originated from stress concentration sites due to the “erosion” of the eclogitization reaction at grain boundaries. Intra-grain shear fractures are ubiquitously observed. Many Cpx and Qtz grains are cut through by fractures. Some of the grains have been completely sheared apart with continuous thin NRBs filling the rupture paths (Fig. 6a, b, 8a). The reaction “notches” appear closely associated with fractures in Cpx and Qtz grains, presumably through stress concentration. Most of the intra-grain fractures are filled with and connected by NRBs across boundaries of different minerals, forming larger-scale fault zones (Fig. 8a, b). Among the three phases in the granulite samples, Plg has the lowest melting temperatures^46^ and Plg melts tend to quench into glasses without dendritic texture^47^. No amorphous materials were found down to 5–10 nm level that might represent quenched glass. Neither were there dendritic crystals in or near the NRBs (Fig. 7a, b; Supplementary Fig. 10).

The EG sample D1787, with eclogitization more advanced than in G samples, also contains macroscopic faults (Fig. 5b). The reaction products form clusters along grain boundaries and within Plg grains (Fig. 6c, d, 8c). Qtz and Cpx grains often have wedge-shaped openings filled with a mixture of Plg and reaction products (Fig. 6d), believed of the same origin as the notches observed in D1996. The wider wedge angles may be due to more advanced syn-deformational eclogitiztion. Thickness of NRBs is greater than that in the G samples with grain sizes up to a few 100 nm (inset of Fig. 6c). Intra-grain ruptures are filled with reaction products (Fig. 8c); larger-scale shear zones are formed by alignment of weakened grain boundaries filled with reaction products (Fig. 8d). Again, no evidence of melting was found (Supplementary Fig. 12).

In contrast to the samples deformed at 1073 K, samples deformed at 1273 K (D1736, D1738) show significant barreling (Fig. 5c) and do not contain faults greater than a few microns in size. This observation, along with delayed AE activity (Supplementary Fig. 8), suggests that at 1273 K the samples were close to the ductile regime. Grain boundaries are dominated by sub-μm sized reaction products, which also appear within Plg grains (Supplementary Figs. 12, 13). Wide-spread reaction rims are observed around grain boundaries (Supplementary Fig. 13a). Some Cpx grains have almost completely transformed into Grt (Supplementary Fig. 13b). Ky needles are dispersed in Plg grains (Supplementary Fig. 13c). At higher magnification, very small cavities (« 1 μm) are also present along grain boundaries (Supplementary Fig. 13b). Fractures in large grains (some of which may be due to pressure release) and cavitation aided grain boundary sliding may be the main cause of observed AEs. No macroscopic faults are present.

## Discussion

The initial eclogitization reaction at 1073 K and 2.5–2.8 GPa (e.g., sample D1996), under the influence of differential stress, may be interpreted as going through primarily the following idealized reactions^46^:

The Ab component in Plg reacts with the Di component in Cpx, forming Omp plus Qtz;

1 $$	\hskip 35pt{\mathrm {CaMgSi}}_{\mathrm {2}}{\mathrm {O}}_{\mathrm {6}}\, \left( {{\mathrm {Di}}} \right)+ x {\mathrm {NaAlSi}}_{\mathrm {3}}{\mathrm {O}}_{\mathrm {8}}\left( {\mathrm {Ab}} \right) \\ 	 {\mathrm { = }}\left( {\mathrm {CaMgSi}}_{\mathrm {2}}{\mathrm {O}}_{\mathrm {6}}{ \bullet x {\mathrm {NaAlSi}}_{\mathrm {2}}{\mathrm {O}}_{\mathrm {6}}} \right)\,\left( {\mathrm {Omp}} \right) + {x}{\mathrm {SiO}}_{\mathrm {2}}\left( {\mathrm {Qtz}} \right){\mathrm {.}}$$

The An component reacts with Di, forming a Ca and Al rich Cpx (Di + Ku) plus Qtz:

2 $$	\hskip 45pt{\mathrm {CaMgSi}}_{\mathrm {2}}{\mathrm {O}}_{\mathrm {6}}\left( {\mathrm {Di}} \right)+ y {\mathrm {CaAl}}_{\mathrm {2}}{\mathrm {O}}_{\mathrm {8}}\left( {\mathrm {An}} \right) \\ 	{\mathrm { = }}\left( {\mathrm {CaMgSi}}_{\mathrm {2}}{\mathrm {O}}_{\mathrm {6}}{ \bullet y {\mathrm {CaAl}}_{\mathrm {2}}{\mathrm {SiO}}_{\mathrm {6}}} \right)\,\left( {\mathrm {Di+Ku}} \right) + {y} \, {\mathrm {SiO}}_{\mathrm {2}}\left( {\mathrm {Qtz}} \right).$$

where Ku stands for kushiroite (CaAl_2_SiO_6_). An breaks down to grossular (Grs) and Ky plus Qtz:

3 $$3\,{\mathrm {CaAl}}_2 {\mathrm {Si}}_2{\mathrm {O}}_8\left( {\mathrm {An}} \right) =	 \,{\mathrm {Ca}}_3{\mathrm {Al}}_2{\mathrm {Si}}_3{\mathrm {O}}_{12}\left( {\mathrm {Grs}} \right) + 2{\mathrm {Al}}_2{\mathrm {SiO}}_5\left( {\mathrm {Ky}} \right) \\ 	+ {\mathrm {SiO}}_2\left( {\mathrm {Qtz}} \right).$$

Ab breaks down to jadeite (Jd) and Qtz:

4 $${\mathrm {NaAlSi}}_3{\mathrm {O}}_8\left( {\mathrm {Ab}} \right) = {\mathrm {NaAlSi}}_2{\mathrm {O}}_6\left( {\mathrm {Jd}} \right) + {\mathrm {SiO}}_2\left( {\mathrm {Qtz}} \right).$$

The solid solution of Omp and Di + Ku is a more general form of Omp less rich in Na. The reaction between Plg and Di-rich Cpx has a positive Clapeyron slope^46^, because it is highly exothermic and accompanied by a ~10% volume reduction^48^. Reaction (3), which occurs at pressures ~0.5 GPa lower than Reaction (4)^46^, takes place within Plg grains producing the wavy NRBs wherein. Reactions (3) and (4) also have positive Clapeyron slopes with even greater volume reduction about 20%^48^. As these reactions proceed under differential stress, NRBs tend to propagate in a runaway fashion due to local increase in temperature, which enhances transformation kinetics. We conducted extensive search for evidence of melting, using high-resolution TEM (HRTEM) and SAED with apertures ~50 nm. No amorphous materials, which may be an indicator of quenched melt, were found at length scales down to ~10 nm (Fig. 7, Supplementary Fig. 12). For D1996 (*P* = 2.8 GPa), the melting point (*T*_m_) of Plg is about 1673 K^49^. Based on the offsets of sheared grains (e.g., Fig. 6a, b), we may estimate the upper bound for the frictional coefficient (*μ*) of the NRBs according to $\Delta T > \mu \sigma _{\mathrm {n}}\frac{D}{{{\mathrm {pc}}\,h}}$, where Δ*T* = *T*_m_−*T* ≈ 600 K, *σ*_n_ the normal stress (=*P*), *D* the “coseismic” slip distance (~20 μm), ρc is the specific heat capacity (~2.5 MPa K^−1^), and *h* the thickness (~1 μm) of the NRBs^50^. According to this relation, which assumes that Coulomb friction law applies at depths and shear-induced heating is adiabatic, *μ* is <0.03. For such low friction coefficient, the NRBs are almost fluid-like.

In EG samples, the reaction products are predominantly Grt, Ky, and Qz, following Reaction (3). The Cpx component in granulite further reacts with the above reaction products forming a Grt phase that contains pyrope (Pyr) and almandine (Alm), in addition to 40–50 mol% Grs. Grain sizes of these phases are slightly larger than those in G samples (Supplementary Fig. 12b). The reduction in flow stress in this sample is attributed to pronounced grain boundary weakening, due to the formation of ultrafine-grained reaction products, analogous to that observed in^51^. Large faults in EG samples are formed predominantly by thickening and coalescing fine-grained reaction bands, which then connect across grains of different minerals, forming macroscopic shear bands (Fig. 8d).

**Fig. 9:**
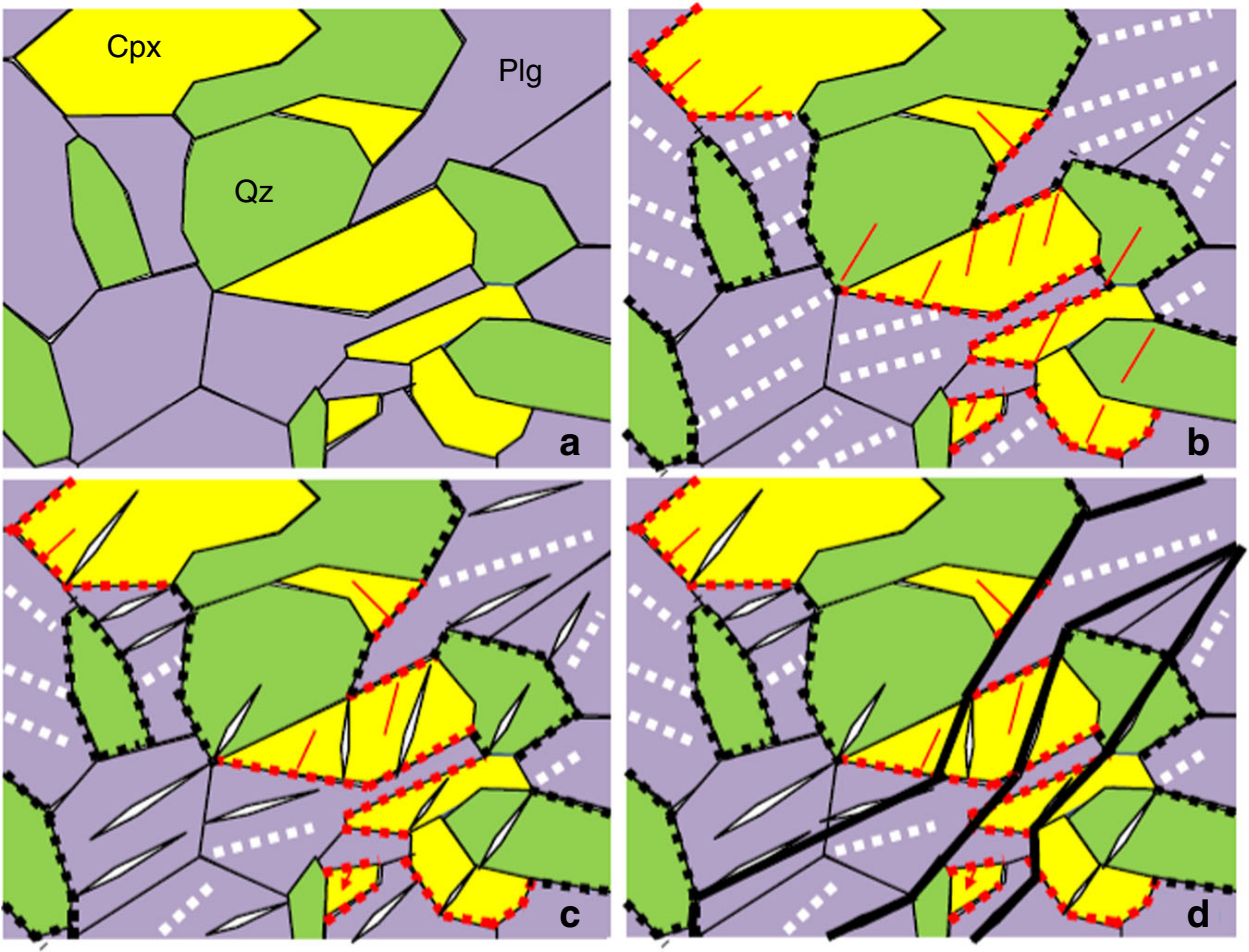
Schematic illustration of the self-organization process of fault zones in metastable granulite deformed in eclogite stability field. **a** Initial microstructure with clean grain boundaries and mineral separation among Plg, Cpx, and Qtz. **b** Reactions (1) and (2) occur along the Plg + Cpx (red dashed lines) and Plg + Qtz interfaces (black dashed lines). Within Plg grains Reaction (3) takes place (white dashed lines). These are the nuclei of NRBs. As reactions proceed, stress concentration sites increase along interfaces, initializing micro-cracks propagating inward individual grains (thin red lines). **c** NRBs grow within Plgs grains and along mineral interfaces, causing shear displacements in Plg grains and grain boundary sliding. Micro-cracks occur in Cpx and Qtz grains. **d** NRBs and micro-cracks connect forming larger fault zones (black lines)

Our 4-D event tracking shows that failure nucleated inside the samples and propagated outward (Fig. 4 and Supplementary Movies 1–3). Microstructural observations on samples with various deformation histories allow construction of the following model of mechanical instability during syn-deformational eclogitization (Fig. 9). An initial granulite microstructure (Supplementary Fig. 5a) is schematically illustrated in Fig. 9a. As the rock is deformed in the eclogite stability field, Reactions (1) and (2) occur along Plg-Cpx interfaces (red dotted lines in Fig. 9b). Reaction (3) occurs within Plg grains (white dotted lines) and also along the Plg-Qtz interfaces (black dotted lines), presumably due to stress concentration. These reactions produce NRBs consisting of ultrafine-grained products and perhaps minute cavities (dotted lines). Latent heat produced by the reactions raises temperature locally, further enhancing rates of reactions. The combined effects result in a transient loss of shear strength along mineral interfaces and in Plg grains. As the reactions proceed, mineral interfaces are further “eroded”, producing more stress concentration sites at Cpx and Qtz grain boundaries, where microfractures initiate (schematically shown as thin red lines in Fig. 9b). NRBs within Plg grains and along mineral interfaces become shear bands, within which shear deformation is concentrated. The reaction products, being ultrafine-grained and fluid-like, fill in microfractures and grain boundaries. Intra-grain shear bands and microfractures propagate across individual grains (Fig. 9c), producing AE events whose magnitudes are primarily controlled by the dimensions of the grains. These microfractures form *en-échelon*-like configurations, which are the nuclei of Riedel-like shears in the synthetic sense^52^. As deformation continues, intra-granular fractures connect across “lubricated” grain boundaries and interfaces, forming larger fault zones (Fig. 9d, thick black lines). Figure 9 only shows growth of fault zones in one general direction. The same argument applies to the conjugated directions. The fault zones are frictional shear in nature and, with increasing deformation, produce antithetic and other accessory Riedel shears as required by geometric and stress/strain compatibility^52^. The sub-mm scaled Riedel-like shears (Fig. 8b) exhibit similar characteristic of fault zones to those observed in larger laboratory test specimens and in the field, from cm to km scales^52^, suggesting that once the length of Riedel shear zones grows to greater than several tens of grain diameters, mineralic heteorogeneity becomes less important, and mechanical behavior is controlled by the properties of the average continuum.

Abundant hydrous mineral epidote was found in pseudotachylites from Bergen Arcs, Norway, suggesting significant amounts of hydrous fluids were present during the formation of pseudotachylites^53^, which are considered indicators of shear-induced melting^38^. In our samples, the small amount of structural water (<280 ppm) was insufficient to stabilize epidote. Furthermore, our microstructure observations suggest that shear instability does not necessarily induce melting. In fact, there is growing evidence, from both field and laboratory, that fine-grained reaction products can significantly affect mechanical behavior of deforming rocks^54^, resulting in reaction softening^55^ or transformational faulting^56^ in the solid state. Dry granulite is another example where mechanical instability is due primarily to grain-size reduction.

Seismic moment magnitudes of the AEs exhibit a very narrow distribution (Figs. 2b, 3b). This is consistent with grain-size controlled microfracturing observed in the microstructure. Such narrow magnitude distribution, however, prevents a meaningful Gutenberg-Richter statistics analysis, and *b*-values cannot be reliably evaluated. In contrast, the large sudden jump in strain around 6000 s (Fig. 2a), corresponding to a ~0.1 mm instantaneous sample shortening, strongly suggests formation of macroscopic faults and is consistent with the long and complex Riedel shears observed in the microstructure (Fig. 5a). Therefore, large events responsible for Riedel shears may have characteristic frequencies outside of the detectability of the narrow band transducers used in this study. Future development of broadband transducers is required to examine scalability issues based on *b*-values.

Both mechanical and microstructural observations indicate that granulite-facies rocks at the onset of eclogitization are seimogenic. At our laboratory strain rate (c. 10^–5^ s^−1^), the temperature at the onset of eclogitization is ~1073 K. Under such temperatures granulite deforms by power law creep within its own stability field^42,43^. Thermokinematic modeling suggests that eclogitization of the metastable Indian lower crust begins at temperatures ~873 (±100) K^57^. Based on *P*-wave velocity anomalies, it has been argued that below ~60 km the lower crust may have reached roughly 30% eclogitization^32^, assuming a homogeneous mixture of granulite- and eclogite-facies minerals. Actual degrees of eclogitization depend on temperature, age, and volatiles and therefore vary within the subducted crust^2,57^. Extrapolating the granulite flow law to 10^−16^ s^−1^ (a representative strain rate in subducted Indian lower crust^58^) and 873 K yields flow stresses on the order of 2–4 MPa, in good agreement with strength models for continental crust and lithosphere^11^, as well as Bouguer gravity and topography data, which infer a long-term effective elastic thickness of 70–120 km for this region^59^. The ~4 MPa crustal strength also provide a broad constraint on earthquake stress drops, which are of similar magnitudes in southern Tibet^60^.

Our results provide a viable micro-mechanism for rupture nucleation and propagation for observed lower-crustal seismicity in southern Tibet. However, mechanisms for the seismicity below the Moho remain unresolved. The unusually large effective elastic thickness of lithosphere indicates that the uppermost mantle must be rigid enough to contribute significantly to the long-term integrated strength of the lithosphere^59^. A recent study shows that at pressures corresponding to depths up to 100 km, shear-banding and localized heating may develop in deforming olivine, resulting in seismogenic faulting^23^. We note that sub-Moho earthquakes in southern Tibet tend to be clustered in close proximity to events in the lower crust (Fig. 1b). Such sub-Moho seismicity may be interpreted as shear localization induced instability triggered by stress heterogeneities produced through strong mechanical coupling between the crust and upper mantle.

## Methods

### Experiments and procedures

Deformation experiments were conducted in a modified Griggs apparatus^42^ with conventional continuous stress and strain measurement at State Key Laboratory of Geological Processes and Mining Resources, CUG (Wuhan), and in a D-DIA apparatus with in-situ AE and X-ray monitoring^39,40^ at beamline 13-BM-D of the GSECARS facilities at the Advanced Photon Source, Argonne National Laboratory. The cell assembly used in the Griggs apparatus is shown in Supplementary Fig. 3.

The overall D-DIA setup is schematically shown in Fig. S4. The D-DIA employs six anvils, four of which are made of tungsten carbide (WC), and two made of sintered diamonds, which are transparent to X-rays, allowing complete diffraction Debye rings of the samples to be recorded on the area detector. Cell assembly used in the D-DIA experiments (Supplementary Fig. 5) is identical to that reported in ref.^39^. Deformation was achieved by advancing the two differential rams, shortening the sample along the vertical (cylindrical) axis. Since the differential rams can be adjusted independently from the main hydraulic ram, differential stress and sample axial strain can be controlled essentially independently from hydrostatic pressure. Pressure and temperature conditions corresponded to the stability fields of both granulite and eclogite-facies (Table 1). For each experiment, hydrostatic pressure was first applied to the sample and maintained at a desired value (the “confining pressure”), and then the sample was heated to a targeted temperature. Deformation was carried out after annealing at the target temperature for 1 h, to minimize effects of cold compression and possible water absorption during cell preparation.

The incident X-ray beam was collimated to 200 × 200 μm by WC slits. A Rayonix SX-165 two-dimensional (2D) detector was used for x-ray diffraction (XRD), with monochromatic radiation at 60 keV (wavelength *λ* = 0.2755 Å). Typical diffraction data collection time was 300 s. Detector orientation relative to the incident beam was calibrated using a CeO_2_ powder standard with the data collection and analysis software Dioptas^61^. Stress analysis was carried out on 2D XRD patterns using the Multifit-Polydefix software developed by S. Merkel (http://merkel.zoneo.net/Multifit/). Possible detector center drift was systematically corrected during the stress analysis by adjustment of the diffraction ring center in Multifit-Polydefix.

Sample axial strains were measured using radiography with a large beam (2.0 × 3.5 mm) by opening up the WC slits. Gold foils were placed on top and bottom of the sample as strain markers. Each radiograph was acquired for 8 s using a Ce-doped YAG scintillator and a PointGrey charge-coupled device (CCD, 1.3 μm/pixel). Sample bulk axial strain was defined as positive under shortening, *ε* = Δ*L*/*L*, where *L* is the sample length. The reference *L*_r_ (in Δ*L* = *L*_r_−*L*) was taken just before starting the deformation in each run, under pressure and temperature. Typical uncertainty in strain measurement was on the order of 0.2%. Throughout a given deformation cycle, diffraction and imaging data were collected alternatively by switching the diffraction/imaging optics. The process was automated with minimal human intervention. The total time required for a pair of strain and stress measurements was about 8 min, which is the time resolution in real-time faulting detection.

### AE monitoring in the D-DIA

The AE setup (Supplementary Fig. 4) is similar to that reported in^39^. Six piezoelectric lead-zirconium-titanate (PZT) transducers (10 mm diameter, 0.5 mm thickness), each positioned on the back end of an anvil, were used to monitor AEs arising from the sample. Resonant frequencies of the transducers were 2–3 MHz. The back end of the anvils was mirror-polished to create a perfectly planar interface. For electrical insulation, each sensor was glued onto a 0.25 mm thick Al_2_O_3_ disk. Six amplifiers filtered analogically (0.3–5 MHz) were used to amplify the acoustic signals (at 60 dB). Two types of acoustic data were acquired. Continuous waveforms were recorded at 10 MHz sampling frequency using a Mini-Richter streamer (Itasca Consulting Group, ltd.) during both cold compression and heating cycles. This type of data records events at all magnitude levels. However, the amount of data is large: a continuous recording for 20 min leads to ~50 GB of data. In order to increase streaming duration over the course of an experiment, only one channel was used for continuous recording. Independently from the continuous mode, AE waveforms were acquired simultaneously in triggered mode from all six transducers each using a digital multi-channel oscilloscope. When the signal was higher than a prescribed trigger level (typically 150 mV) on a given channel, the waveform was recorded. Typical triggered waveforms contained 2000–8000 points at a sampling rate of 50 MHz.

Supplementary Fig. 6 shows an example of AE waveforms recorded. Green and red ticks mark the first arrival peak for each channel. Note that, due to faster wave speeds in diamond, arrivals on the two SD anvils are significantly earlier than in the other four WC anvils. To calibrate travel times through the anvils, a 500-V pulse was sent from one sensor with *P*-wave signals recorded by the opposing transducer. Travel times along the three pairs of anvils were then used to build a velocity model, which is necessary to locate the AEs using differences in arrival times. Travel times within WC anvils were assumed to be equal. Travel times within SD anvils were about 1 µs faster than that in the WC anvils. The AE arrival time differences obtained on each sensor, once corrected for anvil travel times, were interpreted as travel-time difference within the assembly.

### Stress analysis

Under hydrostatic stress, the inter-planar spacing (*d*-spacing) for a given set of lattice planes (*hkl*) in a random powdered sample undergoes identical elastic deformation (lattice strain) regardless of azimuth angle on an area detector, resulting in perfectly circular diffraction Debye rings. Under a macroscopic differential stress, the lattice strain is a function of orientation of the diffracting planes relative to the stress geometry, resulting in pseudo-elliptical Debye rings. The pseudo-ellipticity of powder diffraction rings is a linear elastic function, proxy for the differential stress the polycrystalline sample supports. The diffracting plane azimuth *φ* is defined as the angle between the maximum compression axis and the diffracting plane normal. *φ* = 0° corresponds to planes whose normal is parallel to the maximum compression axis.

Each XRD pattern was integrated over 5° slices of azimuthal angle, resulting in 72 chi files (2*θ* v. intensity spectra), using macros in Fit2D^62^. At any azimuth angle *φ*, simple elastic theory assuming no lattice preferred orientation, no plastic relaxation of stress, and under an axial-symmetric stress field predicts the relation between measured d-spacing *d*_hkl_ and lattice strain *Q*_hkl_ to be^63^

5 $$\frac{{d_{{\rm{hkl}}}\left( \varphi \right) - d_{P({\rm{hkl}})}}}{{d_{P({\rm{hkl}})}}} = Q_{{\rm{hkl}}}\left( {1 - 3{\rm{cos}}^2\left( \varphi \right)} \right)$$

where *d*_*P*(hkl)_ is the d-spacing under isotropic stress (i.e., hydrostatic pressure). For each plane hkl, *d*_hkl_ and *φ* are measured from the XRD pattern (cos *φ* = cos *θ* cos *η*), where *η* is apparent azimuth angle on the detector and *θ* the diffraction angle. *Q*_hkl_ and *d*_P(hkl)_ are extracted by fitting *d*_hkl_ vs. *φ* according to (S1).

Differential stresses *t*_hkl_ were calculated for planes from lattice strains *Q*_hkl_ according to

6 $$t_{{\rm{hkl}}} = 6Q_{{\rm{hkl}}}G_{{\rm{hkl}}}$$

The “effective moduli” *G*_hkl_ were calculated with elastic compliances *S*_ij_ from inversion of the stiffness tensor (*C*_ij_) for Qtz from^64^ with experimentally determined pressure^65^ and temperature derivatives^64^. We used the Reuss (iso-stress) assumption to calculate differential stress for each hkl, believed to be closer to reality than the Voigt (iso-strain) assumption^54^. The flow stress quoted in this work is the unweighted average of differential stresses *t*_hkl_ calculated from the lattice strains of Qtz 110 and 101. This approach may be biased by the availability of diffraction lines and has no physical ground; however it is used in many studies because there is still no complete theory for evaluation of macroscopic stress from powder diffraction data. An additional complexity in our experiments is that the samples contain multiple phases, each having a finite strength, resulting in potentially significant stress heterogeneities. The Qtz peaks used may sense macroscopic stresses differently in different samples at various stages of deformation. Recognizing these complications, the stress values thus obtained are considered an indicator of the relative differential stress levels experienced by the material (i.e., stress drops and increases are more reliable than absolute values), with absolute errors as large as 10–20%.

Unit-cell volumes of Qtz were calculated using the two crystallographic planes of 110 and 101, near the “magic angle” cos^2^*φ* = 1/3 (see Equation S1). Confining pressures were calculated following the third-order high-temperature Birch-Murnaghan equation of state^66^, with 1 atm thermal expansion data for Qtz^67^ and pressure and temperature derivatives of the bulk modulus from^64^ and^65^, respectively. Estimated uncertainties in pressure was about 0.2 GPa.

### AE location using hypoDD

AE events were relocated using the well-developed seismological algorithm hypoDD^44^. We first cross correlated waveforms between events for each channel, using a time window of 6 μs wide starting 1 μs before the first *P*-wave arrival. The CC results were visually inspected. For all six channels, CC coefficients greater than 0.8 could identify similar waveforms and align them correctly. Differential arrival times of event pairs measured by the waveform CC were then used in the hypoDD algorithm to relocate the AE events. The hypoDD algorithm determines relative location and origin time between any two events. In the analysis, *P*-wave velocity of granulite, 6.36 km/s^68^, was used, as the amount of eclogite was less than 10 vol% according to SEM. For each pair of events, differential *P*-wave arrival times of at least four channels were needed. To avoid the situation where all four channels were located in a plane, which would lead to a trade-off between the origin time and relative location in the direction perpendicular to the plane, we required differential *P*-wave arrival times of at least five channels for the pair. For each experiment, events recorded could not be located in one group to satisfy the above requirements. The algorithm divided events in groups of various sizes and determined relative locations of events within each group.

### AE energy evaluation

Triggered AE records were first harvested as discrete events. The relative elastic energy emitted is directly proportional to the area under a given AE waveform^69^. In the present study it was measured as the area under rectified signal envelope (MARSE – Supplementary Fig. 7)^69^. Although this measure does not give absolute energy values radiated by the AE events, it provides useful information on relative energy release.

### Effects of strain rate, stress drop, and temperature

In addition to the length scale difference, laboratory experiments must be conducted at much higher strain rates than geological processes for obvious reasons and, as a result, require much greater differential stresses. Laboratory studies show that granulite deformation follows the power law creep,

7 $$\dot \varepsilon = A\sigma ^ne^{\left( { - Q{\mathrm {/}}RT} \right)},$$

where $\dot \varepsilon$ is strain rate, *A* is a material constant, *σ* the flow stress, *n* the stress exponent, *Q* the activation energy, *T* the temperature (in K), and *R* the gas constant. For mafic granulite^42^, *n* = 3.2, *Q* = 244(35) kJ/mol and for felsic granulite^43^, the corresponding parameters are (no reported uncertainties) 3.1 and 243 kJ/mol, respectively.

Our experiments show that faulting occurs only in samples with a small degree of eclogitization, hence flow properties of such samples are similar to those of pure granulite. Assuming that the subducted Indian lower crust deforms by the same granulite power law mechanism, the required strain rate and flow stress may be estimated from laboratory data using (7), to yield

8 $$\frac{{\dot \varepsilon _1}}{{\dot \varepsilon _2}} = \left( {\frac{{\sigma _1}}{{\sigma _2}}} \right)^n\exp \left( {\left( {\frac{Q}{R}\left( {\frac{1}{{T_2}} - \frac{1}{{T_1}}} \right)} \right.} \right),$$

where subscripts 1 and 2 are parameters for field and laboratory deformation, respectively. Strain rates $\left( {\dot \varepsilon _1} \right)$ in the subducted Indian plate are on the order of 10^−16^ s^−1^ ^58^. Slab thermokinematical modeling^57^ suggests that, at such strain rates, eclogitization of metastable granulite rocks begins around *T*_1_ = 873 K. Our experiments, at strain rate $\dot \varepsilon _2$~ 10^−5^ s^−1^, show that eclogitization at *T*_2_ = 1073 K is seismogenic and that by 1273 K the samples begin losing strength and become ductile. The flow stress *σ*_2_ at *T*_2_ is ~1.5 GPa.

Because of the large uncertainties in *Q* determinations, we use a range *Q* = 200–300 kJ/mol, to examine effects of strain rate, stress, and temperature. With the above parameters, we obtain, from (8), flow stresses of granulite at 873 K and 10^−16^ s^−1^ to be *σ*_2_ = 1.8 MPa for *Q* = 200 kJ/mol and *σ*_2_ = 4.2 MPa for *Q* = 300 kJ/mol. These stress levels, if completely released through seismicity, are in excellent agreement with stress drops for earthquakes with normal and reverse faulting focal mechanisms^60^.

### Electron microscopy

High-resolution transmission electron microscopy (HRTEM) and electron energy loss spectroscopy (EELS) were carried out using Argonne Chromatic Aberration-corrected TEM (ACAT, FEI Titan 80-300ST TEM/STEM) with a field-emission gun and an image corrector to correct both spherical and chromatic aberrations, enabling information limit better than 0.08 nm at an accelerating voltage of 200 kV. High-angle annular dark-field imaging, energy-dispersive X-ray spectroscopy mapping were carried out using a Talos F200X S/TEM (operating at accelerating voltage of 200 kV) equipped with an X-FEG gun and a Super X-EDS system.

## Electronic supplementary material

Supplementary Information

Description of Additional Supplementary Files

Supplementary Movie 1

Supplementary Movie 2

Supplementary Movie 3

## Data Availability

The datasets generated during and/or analysed during the current study are available from the corresponding author on reasonable request.
