# Supplementary material for: Lower-crustal earthquakes in southern Tibet are linked to eclogitization of dry metastable granulite
Source: Nat Commun. 2018 Aug 28;9:3483. doi: 10.1038/s41467-018-05964-1 (PMC6113232; doi:10.1038/s41467-018-05964-1)
Supplement: Supplementary file 1 — Supplementary Information [file 41467_2018_5964_MOESM1_ESM.pdf]

Lower-crustal earthquakes in southern Tibet are linked to eclogitization of dry metastable granulite

Shi et al.

Correspondence to: wang@cars.uchicago.edu

This PDF file includes:

Supplementary Text
Supplementary Figs. 1 to 13
Supplementary Tables 1 to 2

Other Supplementary Materials for this manuscript includes the following:

Supplementary Movies 1 to 3

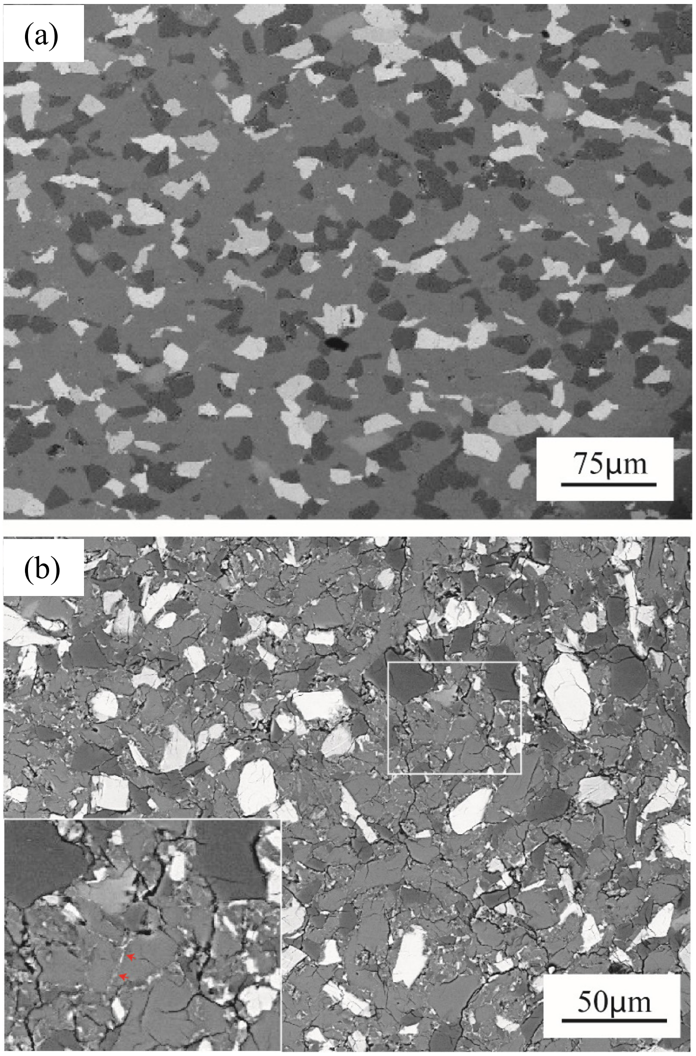

Supplementary Figure 1: **Microstructure of the starting materials.** (a) G sample. White grains: Cpx; grey grains: Plg ; dark grey grains: Qtz. (b) EG sample. Note pervasive “decoration” (red arrow) along the grain boundaries in (b). These “decorating” grains are eclogitization products, predominantly Omp. Inset is enlarged image in the white box. Some Omp “dots” are indicated by the red arrows.

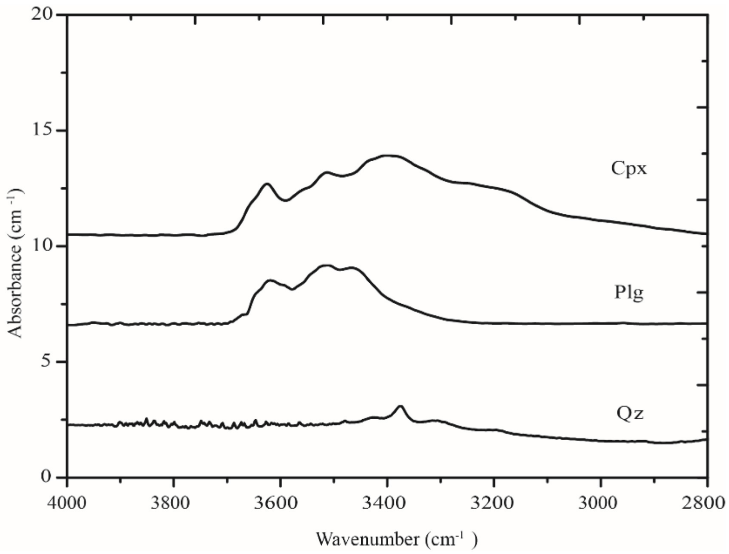

Supplementary Figure 2. Examples of IR spectra of Plg, Cpx, Qtz in starting material "G".

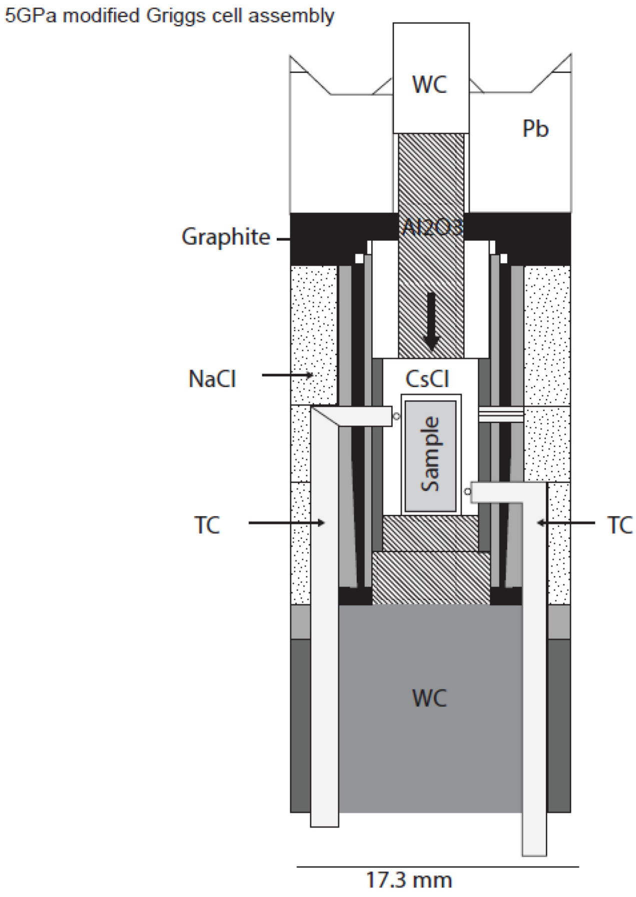

Supplementary Figure 3: **Cell assembly used in the Griggs apparatus.**

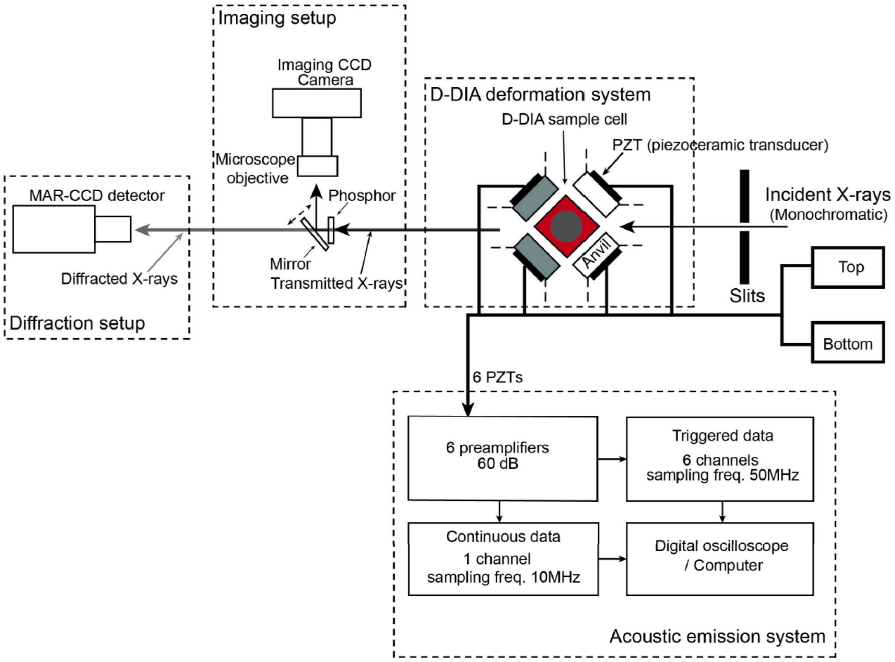

Supplementary Figure 4: **D-DIA experimental setup.** The D-DIA apparatus is shown from the top down, with four horizontal anvils. Top and bottom anvils are not shown. The two anvils in dark grey are sinter-diamond (SD) and the other 4 are tungsten carbide (WC). The imaging optics and diffraction optics were automatically switched during the experiment.

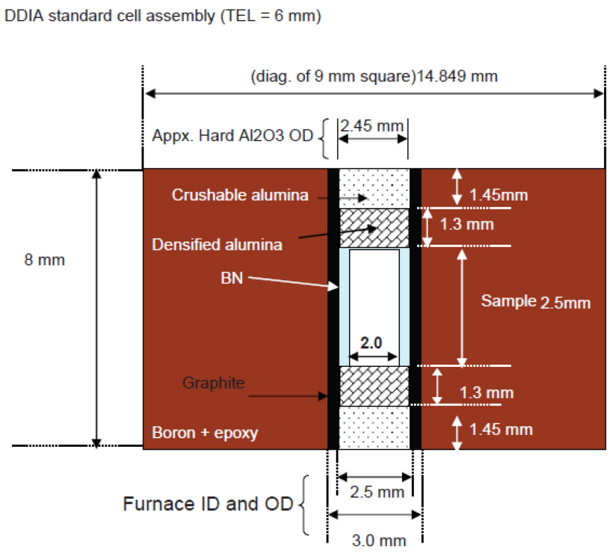

Supplementary Figure 5: Cell assembly design for deformation experiments in the D-DIA.

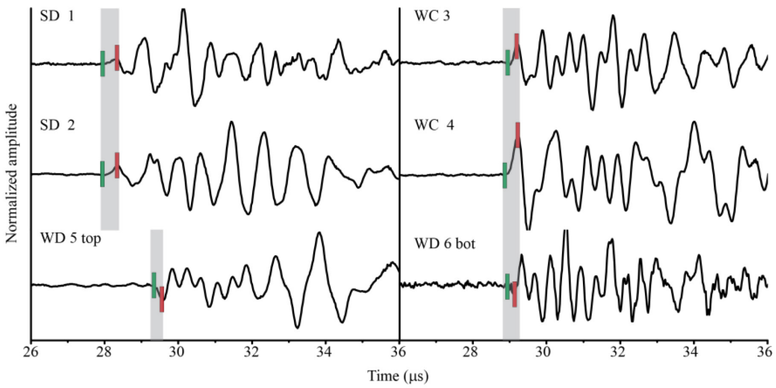

Supplementary Figure 6: **A set of triggered AE waveforms observed in run D1996** (AE record #0116).

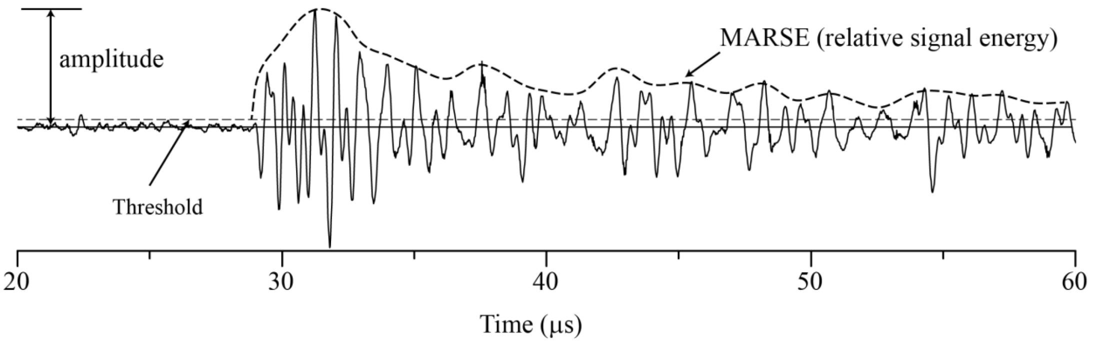

Supplementary Figure 7: Schematic representation showing the definition of MARSE (area under rectified signal envelope). Dashed curve outlines the maxima of the AE waveform.

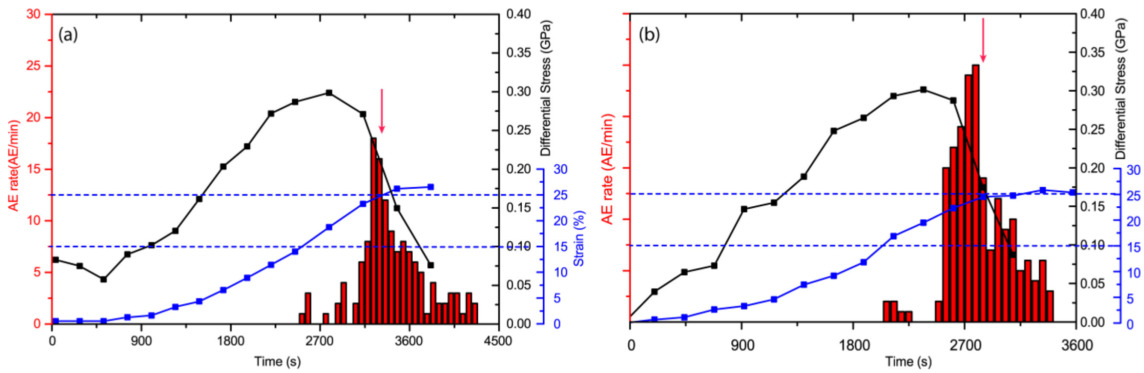

Supplementary Figure 8: AE activity throughout the deformation history for the two EG samples at 1273 K. (a) D1736; (b) D1738. Vertical red arrows point at the time when deformation was stopped. The stress drops beyond the arrows are due to relaxation only.

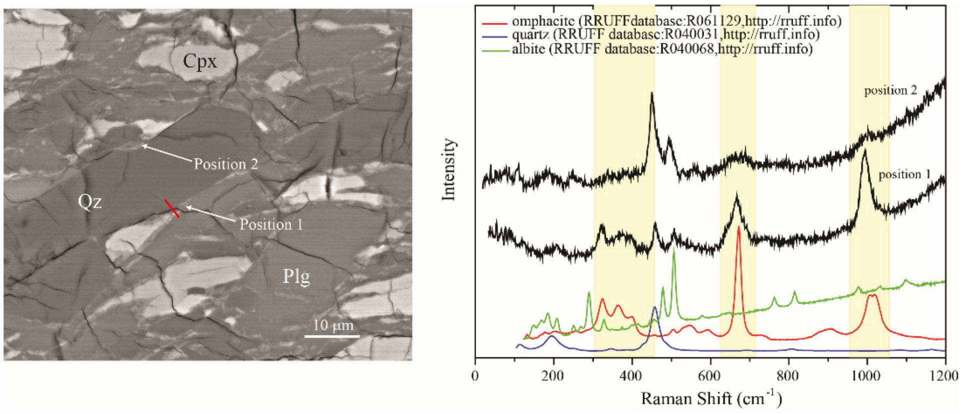

Supplementary Figure 9: **MicroRaman analysis on D1996.** Left figure: SEM of the area analyzed. Right figure: examples of Raman spectra contained. At point 1 (in the brighter shear bands) the reaction products are predominately Omp. Raman spectrum at point 2 is dominated by Qtz and Plg (here the reference is Ab), with small amount of Omp.

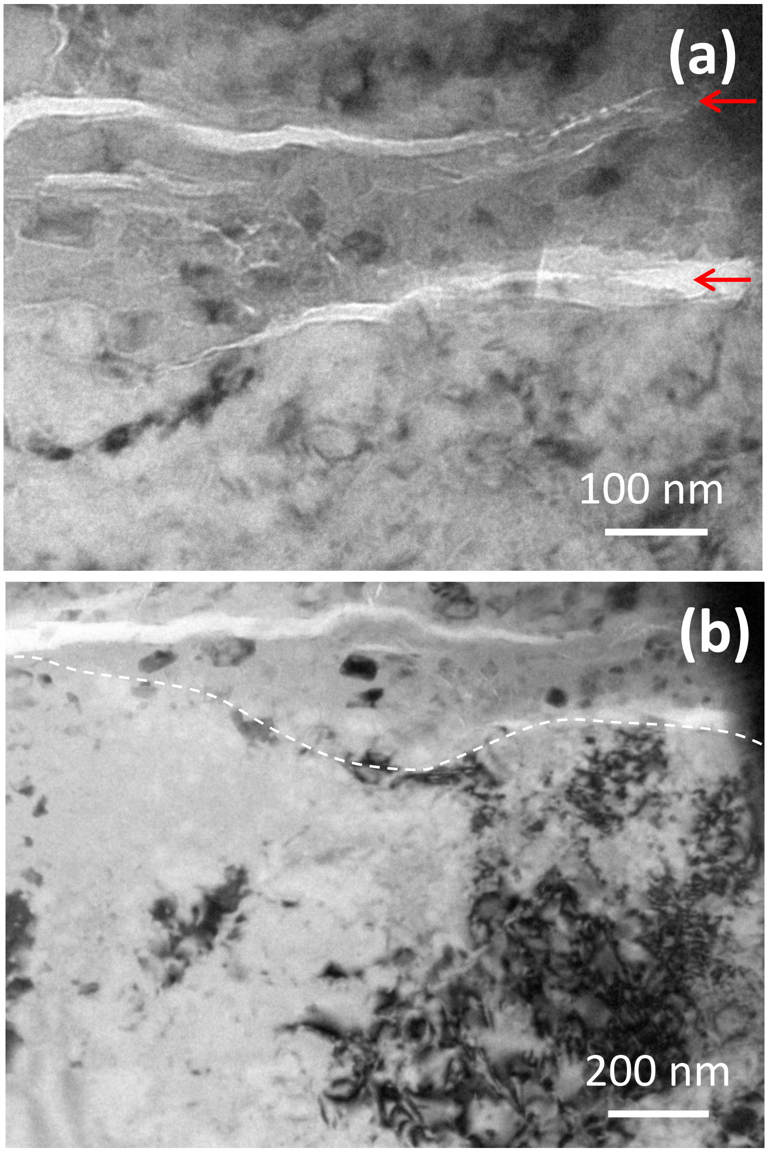

Supplementary Figure 10. Bright-field TEM micrographs showing ultrafine-grained materials in NRBs in sample D1996. (a) An NRB (between the two red arrows) Euhedral, nm-sized grains in the NRB. (b) A fine-grained NRB (above the white dashed curve) in contact with Plg (below) with high density of dislocations.

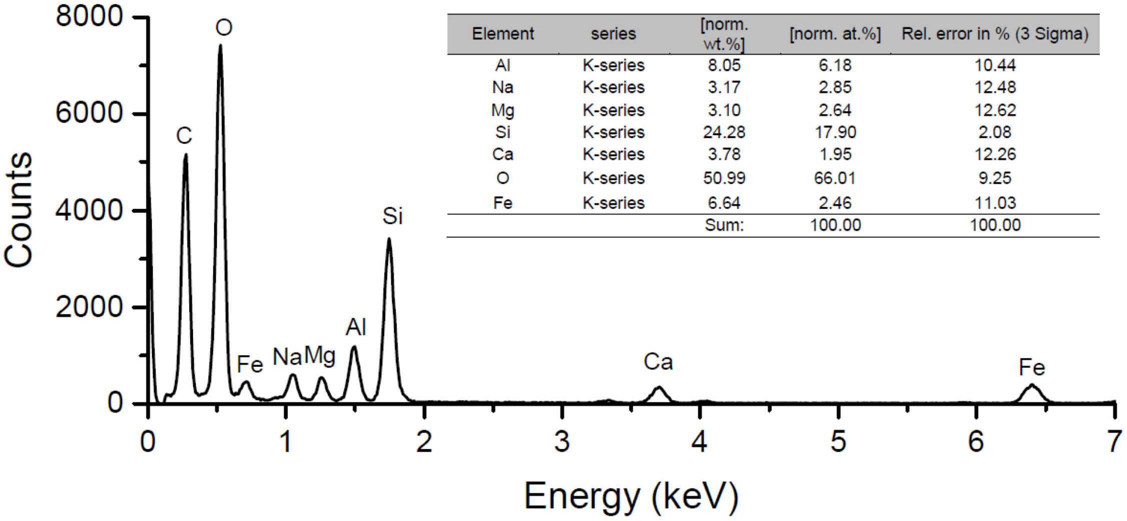

Element	series	[norm. wt.%]	[norm. at.%]	Rel. error in % (3 Sigma)
Al	K-series	8.05	6.18	10.44
Na	K-series	3.17	2.85	12.48
Mg	K-series	3.10	2.64	12.62
Si	K-series	24.28	17.90	2.08
Ca	K-series	3.78	1.95	12.26
O	K-series	50.99	66.01	9.25
Fe	K-series	6.64	2.46	11.03
		Sum:	100.00	100.00

Supplementary Figure 11. EDS spectrum taken from the Na-rich area in Fig 7. Inset table gives the elemental abundance analysis result, yielding an Omp composition approximately $(Ca_{0.2}Na_{0.3})(Mg_{0.3}Al_{0.1}Fe_{0.2})(Al_{0.4}Si_{1.6})O_6$.

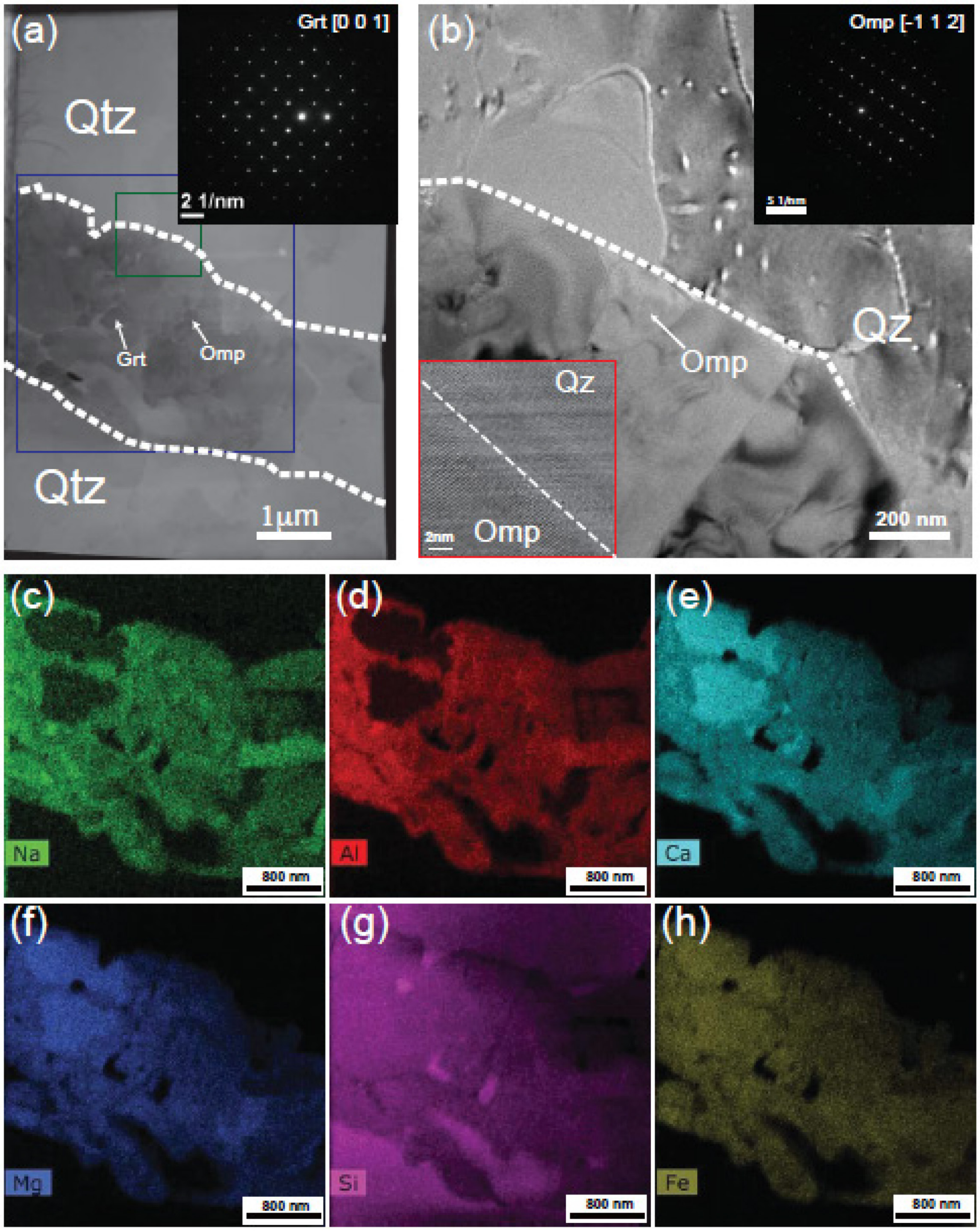

Supplementary Figure 12. TEM micrographs and element distribution mapping along a fault zone in the EG sample D1736. (a) A fault in between two Qtz domains is filled with elcogitization reaction products, in which Grt and Omp have been identified. Fault boundaries are marked by dashed lines. Majority of the grains in the fault are less than 100 nm. Inset SAED pattern is taken from the labeled Grt crystal, along the [001] zone axis. The reaction products are interpreted as fluid-like, permeating micro-cracks and lubricate the fault. (b) Enlarged image of the green box in (a). Dashed line indicates the fault boundary. Inset SAED pattern (upper right) is from the labeled Omp along the [-1 1 2] zone axis. Lower left inset is HRTEM image across the Omp-Qtz boundary, where amorphous material is absent. No evidence of melting can be detected. (c-h) Element distribution of the area outlined by the blue box in (a). Na enriched area is dominated by Omp.

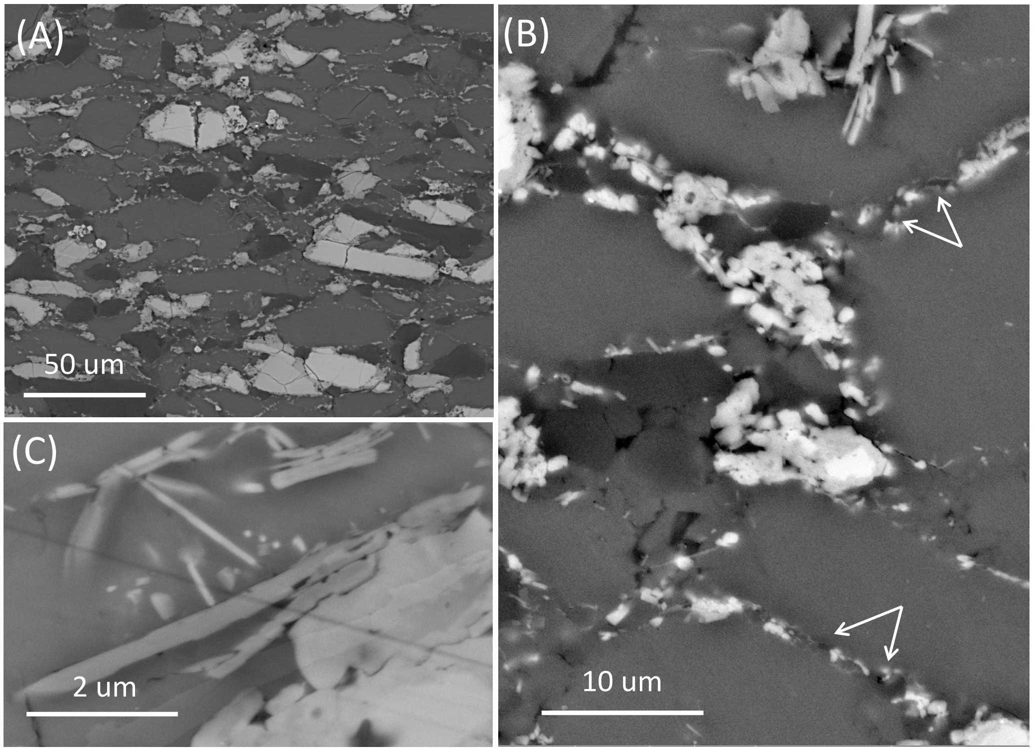

Supplementary Figure 13. Microstructure of an EG sample deformed at 1273 K (D1736). (a): Low-magnification image showing reaction rims around the Cpx and Qtz grains. Intra-granular fractures are filled with reaction products. Some reaction products also appear within Plg grains. (b): Minute cavities are observed along grain boundaries with reaction products (white arrows). These cavities are likely due to the large volume reduction (~20%) associated with reactions exemplified by (S3). (c): Reaction products within a Plg grain, showing Ky as needles and Grt in irregular shapes.

Supplementary Table 1: Compositions of Plg and Cpx in the synthetic granulite *

Mineral	MgO	Al_2O_3	CaO	FeO	SiO_2	MnO	Cr_2O_3	Na_2O	K_2O	TiO_2	Total
**Plg†**	0.01	27.60	10.03	0.13	55.56	0.03	0.04	5.80	0.19	0.02	99.35
**Cpx‡**	11.21	2.71	20.97	13.42	50.29	0.32	0.03	0.50	0.01	0.30	99.73

*: Compositions given as oxides by weight.
†: Resultant Plg solid-solution: $Ab_{0.51}An_{0.48}Or_{0.01}$, where Ab=albite ($NaAlSi_3O_8$), An=anorthite ($CaAl_2Si_2O_8$), and Or=orthoclase ($KAlSi_3O_8$).
‡: Resultant Cpx composition: $Di_{0.66}Fs_{0.22}Wo_{0.12}$, where Di=diopsite ($CaMgSi_2O_6$), Fs=ferrosilite ($FeSiO_3$), and Wo-wollastonite ($CaSiO_3$).

Supplementary Table 2: Structural water contents detected by FTIR in individual minerals.

Mineral	No. IR measurements	Range of water content (ppm)	Average water content (ppm)
**Plg**	13	22-455	135
**Cpx**	11	151-1889	885
**Qtz**	12	50-74	50
