# Supplementary material for: Lower-crustal earthquakes in southern Tibet are linked to eclogitization of dry metastable granulite
Source: Nat Commun. 2018 Aug 28;9:3483. doi: 10.1038/s41467-018-05964-1 (PMC6113232; doi:10.1038/s41467-018-05964-1)
Supplement: Supplementary file 2 — Description of Additional Supplementary Files [file 41467_2018_5964_MOESM2_ESM.pdf]

Description of Additional Supplementary Files:

Supplementary Movie 1: Spatial-temporal distribution of Group-1 events in D1996. Events are color-coded in time as they occurred. All events in this group occurred during the first episode of AE burst (Figs. 2a, b, <4500 s). A well-defined planar distribution can be seen, attributed to the formation of one fault plane.

Supplementary Movie 2: Spatial-temporal distribution of Group-2 events in D1996. All events in this group occurred during the second episode of AE burst (Figs. 2a, b, >4500 s). Events fall into two separate clusters. Events in the off-centered cluster display a planar distribution.

Supplementary Movie 3: Spatial-temporal distribution of Group-1 events in D1787. Events form a "cloud" in the center with poorly defined preferred planar distribution.
